# A Rare Case of Coronary Artery Thrombosis in a Patient With Recently Diagnosed Giant Cell Arteritis: Is Anticardiolipin Antibody Involved?

**DOI:** 10.7759/cureus.8077

**Published:** 2020-05-12

**Authors:** Khine S Shan, Qian Zhang, Sharmila Bisaria, Anubha Tewary

**Affiliations:** 1 Internal Medicine, University of Maryland Medical Center, Baltimore, USA; 2 Internal Medicine, Abington Hospital-Jefferson Health, Abington, USA; 3 Internal Medicine, Cooper University Hospital, Camden, USA

**Keywords:** giant cell arteritis, myocardial infarction, acute coronary syndrome, coronary artery thrombosis, anticardiolipin antibody

## Abstract

Giant cell arteritis (GCA) is an immune-mediated systemic inflammation of large-sized arteries that predominantly affects elderly women. It may be considered as one of the risk factors for acute coronary syndrome (ACS). Moreover, patients with GCA may have increased anticardiolipin antibodies (aCL). However, its relationship with antiphospholipid syndrome (APS) is not clear. We present a case of a unique presentation of GCA with a connection to both ACS and APS. A 76-year-old woman who initially presented to the hospital with a chief complaint of intermittent unilateral headache, blurry vision along with transient aphasia was found to have a biopsy confirmed GCA and subsequently developed left anterior descending artery (LAD) thrombosis. Her hypercoagulability workup was negative except for significantly elevated aCL.

## Introduction

Giant cell arteritis (GCA) is an immune-mediated systemic inflammation of median to large-sized arteries that most commonly affects the aorta and its associated branches. GCA is one of the most common systemic vasculitis that predominantly affects women of northern European origin who are over 70 years old [1]. The incidence of GCA has been reported to be 10.9/100000 [2]. There are reportedly 200/100,000 cases annually in patients over 50 years old [3]. The typical clinical presentations include intracranial symptoms such as unilateral headache, jaw claudication, transient or permanent vision loss as well as extracranial symptoms such as limb claudication and Raynaud phenomenon. The most common complications of GCA include amaurosis fugax that often precedes permanent visual loss and aortic aneurysms formation [3]. GCA diagnosis has the sensitivity of 93.5% and specificity of 91.2% if it meets three or more of the five total criteria: age greater than or equal to 50 years at disease onset, new onset of localized headache, temporal artery tenderness or decreased temporal artery pulse, elevated erythrocyte sedimentation rate greater than or equal to 50 MM/hour, or artery biopsy showing necrotizing arteritis that is characterized by a predominance of mononuclear cell infiltrates or a granulomatous process with multinucleated giant cells [4]. Chronic glucocorticoid therapy is the treatment of choice for GCA [4]. Steroid sparing agents such as methotrexate and tocilizumab (interleukin-6 (IL-6) antibody) have also shown to be effective in achieving remission among patients with GCA [2].

Some studies have shown that patients with GCA are at higher risk for developing cardiovascular events and stroke. However, it is not well known how GCA may cause acute coronary syndrome (ACS). It is also unknown whether an anticardiolipin antibody (aCL) is associated with ACS. We present an interesting case of a 76-year-old woman who was found to have a myocardial infarction (MI) shortly after a temporal artery biopsy confirmed GCA. She was also suspected to have antiphospholipid syndrome (APS) during further hypercoagulability workup.

## Case presentation

Our patient is a 76-year-old Caucasian woman who presented to the emergency department (ED) with three days duration of intermittent right-sided headache and dysarthria. Her past medical history was significant for dyslipidemia and hypothyroidism. Home medications included levothyroxine and ezetimibe-simvastatin. She was hospitalized three days ago for a retro-orbital headache associated with bilateral jaw pain exacerbated by chewing along with diffuse chronic hip and shoulder pain. She was treated with one dose of prednisone in the ED due to the presumptive diagnosis of GCA. However, she was discharged home after her symptoms were resolved. She returned to ED because her headache recurred and was now accompanied by hypertension and dysarthria. 

**Figure 1:**
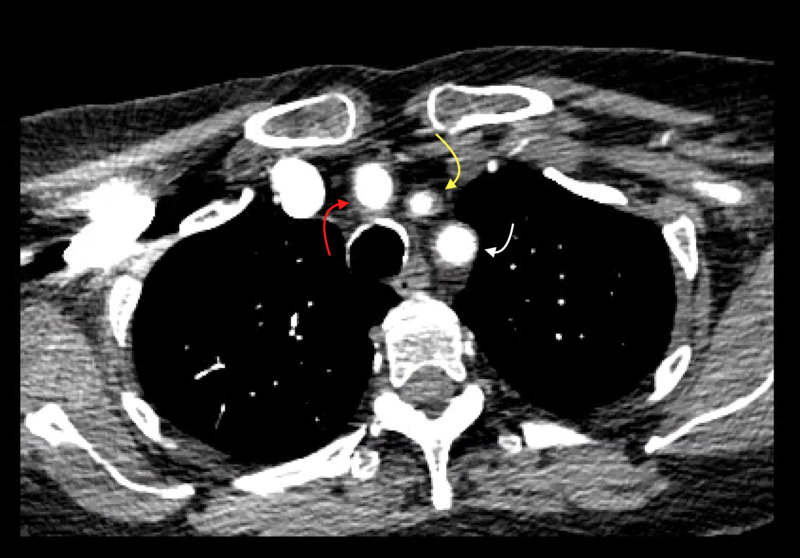
CT angiogram showed wall thickening of the brachiocephalic (red arrow), left common carotid (yellow arrow), and left subclavian (white arrow) arteries suggestive of arteritis

She was afebrile and hemodynamically stable except for blood pressure of 190/90 mmHg upon presenting to the ED. Physical examination was significant for dysarthria but negative for other focal neurological deficits and altered vision. Pertinent labs included: white blood cells 9.6 k/uL, platelet counts 394 k/uL, hemoglobin 13.8 g/dL, erythrocyte sedimentation rate (ESR) 71 mm/hr and negative troponin level. CT head without contrast ruled out acute hemorrhagic stroke. CT angiography of the head and neck showed arterial wall thickening of the brachiocephalic, left common carotid, and left subclavian arteries without evidence of decreased perfusion (Figure 1). She was treated for her blood pressure and also started on prednisone 60 mg daily due to high suspicion for GCA given her clinical presentation and radiological findings. Her headache subsequently improved after the treatment. She remained to be stable overnight.

On day 2-5 of hospitalization, she underwent a right temporal artery biopsy due to high suspicion of GCA. Unfortunately, she was found to have a sudden onset of substernal chest pain within a few hours after the biopsy procedure with electrocardiogram showing anterolateral ST-segment elevation. She was diagnosed with ST-segment elevation myocardial infarction (STEMI) and was taken to the cardiac catheterization lab for an emergent percutaneous intervention. The cardiac angiogram showed no significant stenosis of coronary arteries other than apical left anterior descending artery (LAD) thrombosis with associated severe apical hypokinesis. Subsequently, she was medically managed for an acute myocardial infarction with heparin.

On day six of hospitalization, her temporal artery biopsy result confirmed GCA with pathological findings significant for transmural granulomatous inflammation. Concurrently, the patient’s platelet counts were found to be trending upwards that peaked at 477 k/uL. There were concerns for possible underlying coagulopathy that warranted a lower extremity Doppler study and echocardiogram that ruled out deep vein thrombosis and patent foramen ovale respectively. She had no history of cardiac conditions, thrombophilia or previous clots noted. However, she did have one miscarriage and two stillborn deliveries in the past. Her family history was significant for deep vein thrombosis and pulmonary embolism in two first degree relatives. 

On day 7-12 hospitalization, her vision gradually improved as she continued to receive prednisone therapy. Further thrombophilia workup revealed significantly elevated aCL that were suggestive for APS. Other hypercoagulable workup including homocysteine and anti-beta 2-glycoproteins results were negative. She was discharged on aspirin, metoprolol, and losartan for ACS along with prednisone for GCA. She was also discharged on warfarin for anticoagulation since the usage of direct oral anticoagulants (DOACs) in the setting of coronary thrombosis is currently unestablished. She was advised to follow up in the outpatient setting with Rheumatology and Hematology-Oncology for the continuation of care.

## Discussion

Systemic inflammatory diseases such as rheumatoid arthritis, systemic lupus, and psoriatic arthritis have been associated with an increased risk of cardiovascular disease (CVD) [1]. Similarly, patients may have a higher risk of developing CVD given the fact that GCA is a systemic inflammation that predominantly affects the elderly population. However, GCA as a risk factor for ACS is not well understood. A retrospective population-based study using the Rochester Epidemiology Project medical record linkage system showed that there is no overall increased risk of ACS in patients with GCA [1]. The CVD risk factors including diabetes mellitus and hypercholesterolemia appear to be less frequent with GCA confirmed diagnosis [1]. A systematic review and meta-analysis of case-control and cohort studies by Ungprasert et al. also found that there is no increased coronary artery disease (CAD) risk among patients with GCA [5]. The reason is that patients with GCA have lower initial CVD risk factors such as diabetes and dyslipidemia that might have balanced out the deleterious effect of chronic inflammation on endothelial cells [5]. In addition, the age of patients with GCA is usually in the 7th to 8th decade and complications of chronic inflammation may have not developed before death [5]. 

An observational cohort study of 3408 patients from the United Kingdom showed that patients with GCA have elevated risks of MI, peripheral vascular disease (PVD), and cerebrovascular accidents (CVA) [6]. Furthermore, it suggested that the period immediately after the diagnosis of GCA is associated with a higher relative risk for CVD development [6]. Another population-based retrospective cohort study from Canada which followed older adults with GCA over a ten-year period showed an increased risk for developing CVD including CAD, CVA, PVD, aneurysm formation, and artery dissection [7]. 

It is not well understood how GCA increases CVD risks but may be related to its pathogenesis. Pathogenesis of GCA is thought to be associated with arterial wall infiltration by T lymphocytes, which release interferon-γ that forms multinucleated giant cells by interacting with macrophages, eventually producing growth factors that activate vascular smooth muscle cells [2]. This further induces the proliferation of myofibroblasts causing intimal hyperplasia and luminal narrowing [2]. This correlates with the vascular component of GCA as it causes intracranial related symptoms. However, systemic inflammatory symptoms seen in GCA are usually associated with elevated acute phase proteins like C-reactive protein (CRP) and cytokines IL-6 production by the innate immune system [2].

It can be hypothesized that there is an increased risk of coronary artery stenosis, MI, CVA, and PVD due to vasculitis directly affecting the coronary, cerebral or peripheral circulation respectively. Furthermore, a generalized inflammatory state may accelerate underlying atherosclerosis [8]. There are different mechanisms that GCA could cause CVD including the presence of focal vasculitis that leads to arterial occlusion, reactive thrombocytosis, hyperfibrinogenemia or accentuated platelet response with an increased surface activation and platelet aggregation [9-11]. The presence of chronic inflammation releases cytokines including IL-6 which is an important inflammatory marker in atherosclerosis and acute-phase proteins including the CRP [12]. It may also promote coagulation cascade, and inhibit fibrinolysis resulting in a hypercoagulable state [5]. Accumulation of all these factors can increase the risk of developing a premature CAD as well as a prothrombotic state. 

The aCL antibodies are known to be associated with both arterial and venous thrombosis. However, an association between aCL and GCA is also currently unestablished. There are some studies that showed a higher prevalence of aCL in patients with GCA. It may be elevated in 30% of the study population whereas the prevalence was only 1% in age-matched individuals [13]. The aCL antibodies were found to be present in 20.7% of the patients with GCA as compared with the 2.9% of controls in another study group [14]. However, they may only be the markers of endothelial lesions and chronic inflammation as they do not seem to have thrombotic risk factors in GCA [14]. It was noted that aCL subgroups found in GCA may be reactive antibodies that are targeted directly against antigens revealed by endothelial lesions whereas aCL subgroups found in other autoimmune disorders might have a role in thrombogenesis [13,14]. In contrast, it has also been shown that patients with GCA with elevated levels of aCL at presentation have an increased risk of developing GCA related vascular complications or other vascular diseases such as CVA. Thus, it is suggestive that the aCL antibodies may serve as an independent prognostic marker for future vascular complications in those patients [15]. 

Another theory explained that patients with GCA are at a higher risk for CVDs likely due to the use of steroids which cause dyslipidemia and hypertension [16]. Treatment with high dose glucocorticoids is the standard of care for GCA but unfortunately has atherosclerotic side effects even though it can theoretically lower the risk of CVD due to its anti-inflammatory effects [6]. Thus, it is also important to be aware of possible cardiovascular events soon after GCA diagnosis and treatment.

Even though our patient did not have any significant CVD risk factors or personal history of blood clots, she developed STEMI from coronary thrombosis immediately after temporal artery biopsy. She had no significant coronary stenosis which supports the fact that her MI is likely due to GCA complications rather than atherosclerotic effects. Our literature review revealed a few case reports that described coronary complications of GCA. Andrade et al. described a case report of a 70-year-old man who initially received percutaneous intervention for his LAD stenosis and was suspected to have GCA status post the procedure [12]. He experienced an acute onset of chest pain and was found to have a thrombotic occlusion of a recently implanted LAD artery stent while waiting for the temporal artery biopsy [12]. In contrast, other case reports support the fact that inflammation directly affects coronary arteries. Mednick et al. mentioned a case report of a 76-year-old woman who was being treated with prednisone for GCA but suddenly died due to cardiopulmonary arrest three weeks after the initial presentation [17]. She was found to have evidence of coronary arteritis on autopsy and thus suggested that coronary arteritis can be a cause of MI in patients with GCA in addition to atherosclerotic factors [17]. Similarly, Armellin et al. described a case report of a 76-year-old woman with GCA who was found to have 90% stenosis of the left LAD even though she only had 30% stenosis of LAD two years prior [8]. The superimposed coronary vasculitis inflammation may play a role in this case as there was a rapid progression of coronary occlusion without the presence of other cardiovascular risk factors along with the CT angiography findings of vasculitic changes in other arteries including subclavian, vertebral, femoral arteries [8].

Another case described the involvement of both coronary thrombosis and arteritis. An 83-year-old woman who was on treatment with high dose steroids for GCA was found dead on day three of hospitalization [18]. The autopsy showed an occlusive thrombosis in her LAD and characteristics of coronary arteritis with the fragmentation of internal elastic lamina and inflammatory infiltrate with giant cells in the adventitia [18]. Those cases alert physicians to be aware of possible acute cardiovascular events after the diagnosis of GCA and the need for routine treatment of patients with GCA with a low dose aspirin [6]. Our case further highlights elevated aCL in association with GCA, which could further increase thrombotic risk. Even though not confirmed yet, our patient may have a possible diagnosis of APS due to her history of miscarriage and stillborn deliveries as well as her family history of thrombosis. It is unclear if her elevated aCL might have contributed to her risk of coronary thrombosis in the setting of chronic inflammation from GCA. Therefore, it is important to monitor those patients closely for ACS especially after temporal artery biopsy.

## Conclusions

Our patient’s ACS event post-operatively may be attributed to systemic inflammation, mild thrombophilia or a combination of both factors. It is important to consider whether GCA serves as a risk factor in patients who develop ACS shortly after the confirmed pathological diagnosis. Our case demonstrates a unique presentation of GCA due to its association with APS and ACS. It is important to establish whether a correlation exists between APS, GCA, and ACS through additional research and literature review. The importance of establishing a connection between GCA, APS, and ACS may impact medical therapy, such as whether there is a need to initiate a more aggressive cardiovascular risk modification approach in patients diagnosed with GCA.
